# Outcomes and survival prediction models for severe adult acute respiratory distress syndrome treated with extracorporeal membrane oxygenation

**DOI:** 10.1186/s13054-016-1568-y

**Published:** 2016-12-05

**Authors:** Sacha Rozencwajg, David Pilcher, Alain Combes, Matthieu Schmidt

**Affiliations:** 1 Sorbonne Universités, UPMC Univ Paris 06, INSERM, UMRS_1166-ICAN, Institute of Cardiometabolism and Nutrition, 75651 Paris Cedex 13, France; 2 Assistance Publique-Hôpitaux de Paris, Pitié-Salpêtrière Hospital, Medical Intensive Care Unit, 75651 Paris Cedex 13, France; 3 Australian and New Zealand Intensive Care Research Centre, Department of Epidemiology and Preventive Medicine, School of Public Health, Monash University, Melbourne, Australia; 4 Intensive Care Department, Alfred Hospital, Melbourne, Australia

**Keywords:** Extracorporeal membrane oxygenation, Acute respiratory distress syndrome, Outcome, Predictive survival models, ECMO-related complications

## Abstract

Extracorporeal membrane oxygenation (ECMO) for severe acute respiratory distress syndrome (ARDS) has known a growing interest over the last decades with promising results during the 2009 A(H1N1) influenza epidemic. Targeting populations that can most benefit from this therapy is now of major importance.

Survival has steadily improved for a decade, reaching up to 65% at hospital discharge in the most recent cohorts. However, ECMO is still marred by frequent and significant complications such as bleeding and nosocomial infections. In addition, physiological and psychological symptoms are commonly described in long-term follow-up of ECMO-treated ARDS survivors. Because this therapy is costly and exposes patients to significant complications, seven prediction models have been developed recently to help clinicians identify patients most likely to survive once ECMO has been initiated and to facilitate appropriate comparison of risk-adjusted outcomes between centres and over time. Higher age, immunocompromised status, associated extra-pulmonary organ dysfunction, low respiratory compliance and non-influenzae diagnosis seem to be the main determinants of poorer outcome.

## Background

**Fig. 1:**
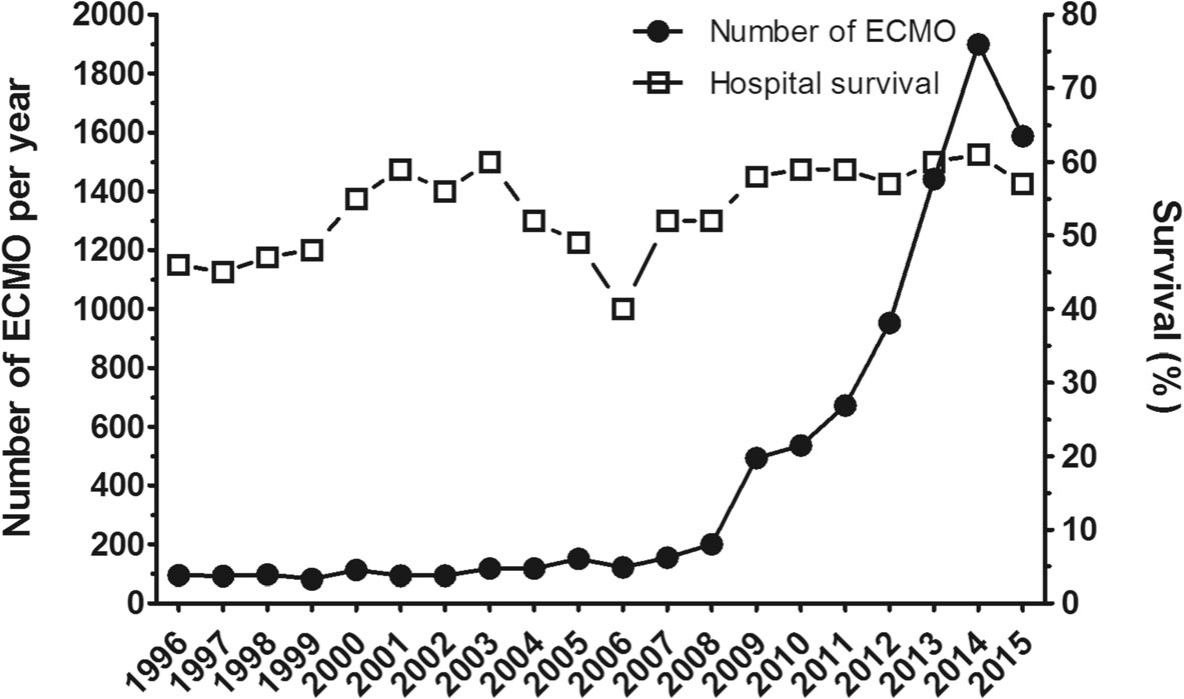
Number of annual adult respiratory cases treated by venovenous ECMO from 1996 to 2015 and the relative hospital survival rate. Adapted from the ELSO ECLS Registry Report [3]. *ECMO* extracorporeal membrane oxygenation

Extracorporeal membrane oxygenation (ECMO) is considered a therapeutic option for patients with severe acute respiratory distress syndrome (ARDS) with refractory hypoxemia or unable to tolerate volume-limited strategies [1, 2]. Use of ECMO has been growing exponentially in the last decade [3], encouraged by promising results from the multi-centred randomized controlled trial CESAR [4] and benefits described during the influenza A(H_1_N_1_) pandemic. In addition, major progress in technology (e.g. smaller devices, heparin-coated circuits, biocompatible membranes, dual lumen cannulae) [5] and network organization, with referral centres and mobile ECMO teams available 24/7, have both contributed to exponentially increase the use of ECMO (Fig. 1). However, despite these improvements, ECMO is still marred by a high rate of complications such as bleeding, thrombosis and nosocomial infection [6–8]. Moreover, ECMO-treated survivors exhibit significant rates of long-term neuro-psychological and/or physical impairment [7]. To date, most of the severe ARDS patients are either referred to ECMO referral centres [4, 9] or cannulated in a distant hospital by a mobile ECMO team [4, 10, 11]. Because this therapy is costly and exposes patients to significant complications, a number of prediction models have been developed recently to help clinicians identify patients most likely to survive once ECMO has been initiated and to facilitate appropriate comparison of risk-adjusted outcomes between centres and over time. This review will describe actual short-term and long-term outcomes of patients with severe ARDS treated with ECMO and summarize the characteristics and performance of published survival prediction models.

## Outcomes of severe ARDS patients with and without ECMO

### Outcomes of severe ARDS treated with “conventional” management

The past two decades have seen significant progress in ARDS management. A more accurate definition has been proposed [12] and major progress has been achieved in understanding the ARDS pathophysiology [13–15] and ventilator-induced lung injury [16, 17]. In addition, protective-lung mechanical ventilation [18] and adjuvant therapies such as prone positioning [19] and neuromuscular blockers [20] have contributed to improvements in overall ARDS mortality. Despite this, the pooled mortality of ARDS (covering all levels of ARDS severity) remains high, even more so in observational studies (48.2%) than in randomized controlled trials (37.5%) [21]. The mortality for severe ARDS is higher still, at 50% [12, 22, 23]. In addition, the burden of ARDS is still perceptible years after ICU discharge, with notable impairment of quality of life [24]. Reported long-term sequelae include ICU-acquired weakness, exercise limitation, frozen shoulders, vocal-cord dysfunction or recurrent reactive airways disease which may contribute to social isolation, psychological morbidity and sexual dysfunction [24]. In a large cohort of 109 patients with ARDS, 51% of patients reported at least one episode of depression and/or severe anxiety within 5 years of follow-up [24]. Nevertheless, 77% of patients returned to work; almost all to their original work 5 years after ICU discharge.

### Outcomes of ARDS treated with venovenous ECMO

Outcomes of patients with ARDS on ECMO have improved steadily over a decade (Fig. 1) thanks to the progress of the devices [5] and better prevention of ECMO-related complications such as bleeding.

The first large international multicentre database on ECMO for severe ARDS was provided by Brogan et al. [25] using a registry issued from a collaborative international network (Extracorporeal Life Support Organization (ESLO)). The data, collected between 1986 and 2006, covered 1473 patients with a median age of 34 years, 78 of whom were treated with venovenous ECMO (VV-ECMO) with a median time of support of 154 hours. They reported an all-cause mortality of 50%. Risk factors associated with a poorer outcome were advanced age, days on mechanical ventilation prior to ECMO and decreased patient weight. These results were relatively consistent with the CESAR trial [4], which reported 63% survival without severe disability at 6 months. In this trial, conducted between 2001 and 2006 in the United Kingdom, 180 patients with severe ARDS were either randomized into ECMO (after transfer to a referral “ECMO centre”) or to conventional management at the referring hospital. These patients suffered from severe and potentially reversible ARDS. Their median age was 40 years (mean APACHE II 20), with a primary diagnosis of pneumonia in 66%. The same year, Australia and New Zealand Extracorporeal Membrane Oxygenation (ANZ ECMO) [26] reported excellent results with a cohort of influenza A(H_1_N_1_)-related ARDS patients. They reported 78% of patients weaned from ECMO and 71% ICU discharge survival despite extreme severity before cannulation (median lowest PaO_2_/F_I_O_2_ ratio 56 mmHg, pH 7.2, PaCO_2_ 69 mmHg and modified acute lung injury score of 3.8). These results should, however, be interpreted with caution because influenza A(H_1_N_1_)-related ARDS has a better prognosis than other causes of ARDS [27, 28]. More recently, Schmidt et al. [6] reported the outcome of 140 patients from three French ICUs. Ninety-five per cent of patients received VV-ECMO with a median time between intubation and ECMO cannulation of 5 (1–11) days. Bacterial pneumonia was the main cause of ARDS (45%). Influenza A(H_1_N_1_)-related ARDS was noted in 26%. Survival rates were respectively 64% and 60% at ICU discharge and 6 months. A cohort of 2355 patients extracted from the international ELSO registry [27] has also been studied recently. ECMO therapy was initiated after a median of 57 hours of mechanical ventilation, with 49% of patients receiving neuromuscular blocker agents, 20% inhaled nitric oxide and 10% high-frequency oscillatory ventilation. Fifty-seven per cent of patients were alive at hospital discharge after a median of 170 hours on ECMO.

### Long-term outcome

The few studies of long-term outcome after ECMO are described in Table 1. The frequent use for young adults with no pre-existing co-morbidities should foster clinicians to measure long-term impact of this therapy. The long-term effects of ECMO have been evaluated broadly in three areas: respiratory function; psychological impairment; and quality of life.

**Table 1:** Studies relating long-term outcomes after ECMO for severe ARDS

Study	Cohort enrolment	Total population	Follow-up		Primary outcome	Long-term outcomes
			population	Median time		
Peek et al.[4]	2001–2006	68	52	6 months	Death or severe disability at 6 months	Lung function evaluated with PFT, overall health status, HRQoL, depression and anxiety symptoms
Lindén et al.[29]	Before 2009	37	21	26 (12–50) months	Pulmonary morphology (CT scan)	Lung function (PFT), pulmonary symptoms (SGRQ)
Hodgson et al.[33]	2009–2011	34	15	9 (8–19) months	HRQoL (SF-36)	Related ECMO complications, survival, discharge destination, return-to-work status
Luyt et al.[31]	Winter 2009	67	12	12 months	HRQoL (SF-36)	Symptoms and activities since hospital discharge, weight and muscle-strength testing, lung morphology (CT scan), anxiety and depression (HAD scale), symptoms of PTSD (IES)
Schmidt et al.[6]	2008–2012	140	67	17 (11–28) months	Factors associated with death at 6 months	HRQoL (SF-36 score), pulmonary symptoms (SGRQ), anxiety and depression (HAD scale), symptoms of PTSD (IES)
Li et al.[30]	2009–2012	29	8	12 months	Pulmonary morphology	

*ARDS* acute respiratory distress syndrome, *ECMO* extracorporeal membrane oxygenation, *HAD* hospital anxiety and depression, *HRQoL* health-related quality of life, *IES* Impact of Event Scale, *PFT* pulmonary function tests, *PTSD* post-traumatic stress disorder, *SF-36* Medical Outcome Short-Form, *SGRQ* St George’s Respiratory Questionnaire

Post-ECMO respiratory impairment can be assessed in three domains: lung capacity assessed by lung function tests; parenchymal changes observed on imaging; and respiratory symptoms. In the CESAR trial [4], lung function tests, performed 6 months post ECMO, indicated relatively preserved lung capacity (forced vital capacity 79.6% predicted, peak expiratory flow rate 54.5% predicted) and were no different to the conventional management group. Similarly, Lindén et al. [29] reported lung function measured at varying time points at least 1 year post ECMO in a cohort of 21 survivors of bacterial pneumonia-related ARDS treated with ECMO. They described slightly impaired lung function with a mildly obstructive pattern (forced expired volume at 1 second < 80%). Measurement of SpO_2_ during exercise tests was low in 43% patients and a reduced DLCO (70% of predicted value) was noted in 65%. In addition, radiological changes compatible with interstitial fibrosis were reported in 76% of the population. In the study by Li et al. [30], 15 patients underwent 1-year follow-up with repeated computed tomography after severe ARDS requiring VV-ECMO. Eighty-seven per cent of patients exhibited similar changes, with more severe damage distributed in the ventral region. In the context of influenza A(H_1_N_1_)-related ARDS, similar findings were reported by Luyt et al. [31]. Lung function tests on 67 patients demonstrated a mild impairment of lung-diffusion properties (DLCO below the fifth percentile of normal values) in both ECMO and non-ECMO survivors with no difference between both groups. No obstructive lung disease was noted and arterial blood gases at rest and after exercise were within normal ranges. However, 75% of patients in the ECMO group suffered from moderate dyspnoea during strenuous exercise at 1 year [31]. Lastly, it is notable that most patients in both groups had returned to work, and one-third practised sport regularly [31].

Health-related quality of life (HRQoL) evaluation assesses both the physical and psychological impact of ECMO among survivors. Lindén et al. [29] first described HRQoL in their cohort of 21 long-term survivors of severe ARDS and ECMO, focusing on the respiratory symptoms, and showed higher scores on the St George’s Respiratory Questionnaire (SGRQ) than normal values, indicating subjective respiratory problems with an impact on daily life. These findings contrast with the CESAR trial where equivalent SGRQ scores were reported in both groups. Most of the studies used the 36-Item Short-Form Health Survey (SF-36) [32] to assess HRQoL. The physical domain scores of the SF-36 reported mobility limitation or self-care restriction in about 20–30% of survivors [4, 31, 33], which may mostly be due to ICU-acquired limb weakness, considered “slight to moderate” when compared with age-matched and sex-matched population controls [6, 31]. Psychological impairment may also jeopardize long-term quality of life of ECMO survivors. Others domains of the SF-36 evaluate vitality, social functioning and emotional status. Data regarding psychological impact of ECMO for ARDS survivors are scarce. However, they were globally impaired when compared with age-matched and sex-matched population controls [4, 6]. These data were consistent with those of Hodgson et al. [33], who reported a 27% decrease in SF-36 mental component scores in ARDS patients who received ECMO. Finally, 25–34% of ECMO patients reported long-term anxiety and depression symptoms, with 15% considered at risk of post-traumatic stress disorder [4, 6]. These results were similar to those reported in other post-ICU studies [34–36].

In conclusion, HRQoL seems to be significantly impaired after ECMO for severe ARDS. This must be interpreted with caution, however, because it may be attributable to the patient’s ICU length of stay and underlying disease rather than to ECMO itself. HRQoL data showing no difference between ECMO and non-ECMO severe ARDS patients tend to confirm this hypothesis [33].

### ECMO-related complications

The two most important and commonly described ECMO-related adverse events are bleeding and nosocomial infection.

Bleeding occurs in about 20% of patients on ECMO with various degrees of severity (i.e. cannula haemorrhage, spontaneous epistaxis, gastrointestinal or intra-cranial bleeding, etc.) [37, 38]. The main mechanisms are anticoagulation, thrombocytopenia and coagulation factor consumption. ECMO circuits are also responsible for impaired platelet function [39] and biological acquired von Willebrand syndrome (AVWS) [40, 41]. However, a study by Abrams et al. [42] reported that severity of critical illness and platelet count at the time of cannulation, rather than ECMO duration, were the best predictors for development of severe thrombocytopenia while receiving ECMO for respiratory failure. Consumption of red blood cells has been reported as approximately 1 unit per ECMO-day while 17% of patients underwent surgery for bleeding issues [43]. Application of a restrictive transfusion policy on ECMO is possible by implementing a lower aim for systemic anticoagulation, a fixed transfusion threshold of 7 g/dl and auto-transfusion during decannulation [44].

Nosocomial infections are also very frequent in ECMO patients. Their incidence varies widely from 11.7 to 64% [1, 45, 46], equivalent to 11.9–75.5 infections/1000 ECMO-days. However, these data are lacking in the specific VV-ECMO population. Among these infections, the two most common were bloodstream infections and ventilator-associated pneumonia with a median of 15/1000 ECMO-days and 4/1000 ECMO-days respectively when pooling several studies [46–49]. Duration on ECMO and patient’s severity were independent risk factors. One should note that the definition of “ECMO-related infection” and the diagnostic techniques for cannula-related infection are not consistent and may account for differences observed between different studies. In addition, antibiotic prophylaxis, routine bacterial surveillance and continuous antibiotics are frequently used in ECMO centres despite no evidence of their benefit [50].

Neurological events occurred frequently in patients on VV-ECMO. Among 135 consecutive patients who had received VV-ECMO, 18 (15 assessable) developed cerebral complications on ECMO: cerebral bleeding in 10 patients (7.5%), ischemic stroke in three patients (2%) or diffuse microbleeds in two patients (2%). Intracranial bleeding, the most frequent complication, occurred early and was associated with higher mortality. Because intracranial bleeding was independently associated with rapid hypercapnia decrease, ECMO onset should be avoided in this situation, but its exact role remains to be determined [51].

Other ECMO-related complications include thrombosis, especially deep vein thrombosis in the cannulated vessels following ECMO. The incidence was estimated at 8.1/1000 cannula-days and routine venous Doppler ultrasound following decannulation in the VV-ECMO population has been advocated [8]. In addition, haemolysis is commonly observed during VV-ECMO. A recent study of 207 paediatric patients with ECMO reported at least one episode of haemolysis in 66% patients. Although haemolysis is frequently considered minor, these patients were more likely to experience a longer ECMO run and require more blood products. After controlling for age, weight, paediatric index of mortality and diagnosis, patients with severe haemolysis were more likely to die in the ICU and in hospital (odds ratio (OR) 6.34, 95% confidence interval (CI) 1.71–23.54; *p* = 0.006) [52]. In adults, further data are needed to investigate the causes of haemolysis on ECMO and to elucidate its influence on morbidity and mortality.

## Survival prediction models

### Objectives of these scores

Because of the significant numbers of ECMO-related complications, the high rates of long-term physical and psychological impairment, and the human and financial cost, identifying specific populations who could benefit most from this therapy is crucial. All of these scores have been derived only from populations already on ECMO. As such, they should be considered most appropriate for predicting who will survive once ECMO has been initiated, comparing outcomes between units and over time, or helping inform clinicians, family members and even occasionally patients themselves of likely outcomes. Without a population of patients who did not receive ECMO, none of the scores so far described can be considered directly applicable for choosing which patients should or should not receive ECMO. However, given the fact that most of the predictive variables described have been recorded during the immediate pre-ECMO period, it is likely that the same factors which predict survival in populations who are on ECMO may also be helpful to select patients for consideration of ECMO.

### Survival prediction scores

**Fig. 2:**
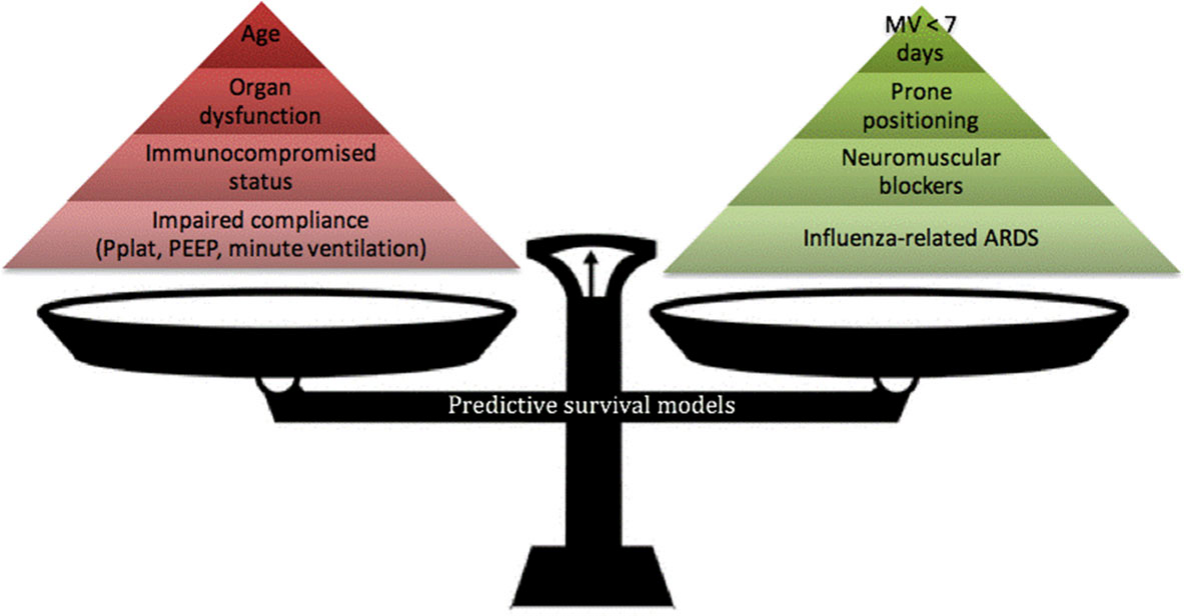
Pre-ECMO factors associated with mortality on VV-ECMO according to published predictive survival models. *Red pyramid*, risk factors; *green pyramid*, protective factors: the higher the factor, the heavier impact on mortality according to published predictive survival models. *ARDS* acute respiratory distress syndrome, *MV* mechanical ventilation, *Pplat, *plateau pressure *PEEP* positive end-expiratory pressure

Over the past 3 years, seven different pre-ECMO survival prediction scores have been published [6, 27, 28, 53–56]; the characteristics of these scores are summarized in Table 2 and Fig. 2. The risk factors taken into account in these models can be divided into four major determinants: demographic characteristics; organ dysfunction; characteristics and management of respiratory failure; and initial diagnosis.

**Table 2:** Survival predictive models for patients on VV-ECMO for ARDS

Score	Population	Number of patients	Number of centres	Cohort enrolment	Pre-ECMO items		Internal validation’s AUROC	External validation’s AUROC
ECMOnet score: Pappallardo et al. [53]	A(H1N1) influenza-related ARDS	60	14	Winter 2009	1. Pre-ECMO LOS 2. Bilirubin 3. Creatinine	4. Haematocrit level 5. Mean arterial pressure	0.86	0.69^a^, 0.60^b^
PRESERVE score: Schmidt et al. [6]	Severe ARDS	140	3	2008–2012	1. Age 2. Body mass index 3. Immunocompromised 4. SOFA score	5. Days of MV 6. Prone positioning 7. PEEP 8. Plateau pressure	0.89	0.68^b^, 0.75^c^
RESP score: Schmidt et al. [27]	Acute respiratory failure	2355	280	2000–2012	1. Age 2. Immunocompromised 3. Days of MV 4. Diagnosis 5. Central nervous system dysfunction 6. Acute associated (non-pulmonary) infection	7. Neuromuscular blockade agents 8. Nitric oxide use 9. Bicarbonate infusion 10. Cardiac arrest 11. PaCO_2_ 12. Peak inspiratory pressure	0.74	0.92^d^, 0.81^c^
Roch et al. [28]	ARDS brought to a referral centre	85	1	2009–2013	1. Age 2. SOFA score 3. Influenza		0.80	No
Enger et al. [54]	ARDS	284	1	2008–2013	1. Age 2. Immunocompromised 3. Minute ventilation	4. Haemoglobin 5. Lactate	0.75	No
Liu et al. [55]	ARDS	38	1	2009–2014	1. Barotrauma^e^ 2. Underlying lung disease		–	–
VV-ECMO mortality score: Cheng et al. [56]	Severe ARDS	116	1	2007–2015	1. Immunocompromised 2. SOFA score 3. Days of MV		0.76	No

^a^Validation in a cohort of 74 patients with A(H1N1) influenza-induced ARDS

^b^Validation in the cohort of Enger et al. [54]

^c^Validation in the cohort of Kleinzing et al. [60].

^d^Validation in the PRESERVE cohort of Schmidt et al. [6]

^e^Barotrauma prior to ECMO was defined as follows: pneumothorax, pneumomediastinum, pneumatoceles or subcutaneous emphysema

*ARDS* acute respiratory distress syndrome, *AUROC* area under receiver operating characteristic curve, *ECMO* extracorporeal membrane oxygenation, *LOS* length of stay, *MV* mechanical ventilation, *PEEP* positive end-expiratory pressure, *RESP* Respiratory Extracorporeal Membrane Oxygenation Survival Prediction, *SOFA* Sequential Organ Failure Assessment, *VV* venovenous

### Demographic characteristics

In all models but ECMOnet and the two most recent published scores [6, 27, 28, 54], age was an independent risk factor. In the PRESERVE and Roch et al. scores [27, 28], being younger than 45 years old was associated with a better prognosis, while a major mortality risk was described for patients over 60 years of age. Immunocompromised status was consistently associated with a poorer outcome in four out of the seven models [6, 27, 54, 56]. For instance, chronic immunosuppression was associated with increased mortality both in Enger et al.’s [54] score (OR 2.6, 95% CI 1.3–5.2) and the VV-ECMO [56] mortality score (OR 2.9, 95% CI 1.1–7.9). Liu et al. [55] found an underlying lung disease (i.e. COPD, interstitial lung disease and lung cancer) to be an independent risk factor for mortality (OR 12.2, 95% CI 1.2–122.2; *p* = 0.033). In no other models were co-morbidities such as chronic organ dysfunction or diabetes identified as associated with poorer outcome. However, there were so few patients with these conditions that it is difficult to raise any conclusion about their impact on outcome.

### Acute organ dysfunction

The number of pre-ECMO organ dysfunctions is unsurprisingly a significant predictive factor. In the Roch et al., PRESERVE, Enger et al. and VV-ECMO mortality scores [27, 28, 54, 56] the SOFA score was used as an organ failure surrogate, whereas mean arterial pressure, serum creatinine, bilirubin and haematocrit levels were used in the ECMOnet score [53]. Lastly, pre-ECMO central nervous system dysfunction was associated with a poorer outcome in the RESP score [27]. In recent retrospective cohorts, SOFA score > 15 was constantly associated with higher mortality [56–58]. However, it is worth noting that pre-ECMO neurological status assessed by the Glasgow Coma Scale score is frequently difficult to evaluate in these patients due to high-dose sedative infusion, making reliability of this neurological SOFA score section questionable [28].

### Characteristics and management of respiratory failure

Management of mechanical ventilation and adjuvant therapies for severe ARDS have greatly evolved during the last decade [18–20, 22, 23]. Amongst the patient cohorts from which scores have been developed, there was evidence of variation in pre-ECMO management, which influenced survival. For instance, only 49% of patients received pre-ECMO neuromuscular blockade in the RESP study [27] compared with all patients in Roch et al.’s cohort [28]. Pre-ECMO nitric oxide and prone positioning were used, respectively, in 16 and 29% of patients in the ECMOnet study [53] vs 90 and 60% in the PRESERVE cohort [6]. Despite the variation in reported use of pre-ECMO adjuvant therapies, where these have been reported, the studies have demonstrated both prone positioning and provision of neuromuscular blockade to be associated with improved survival. These findings are consistent with non-ECMO literature [6, 27]. Duration of mechanical ventilation pre ECMO over 7 days has been significantly associated with a poor outcome in the RESP, the PRESERVE and the VV-ECMO mortality scores [6, 27, 56]. Interestingly, although hypoxemia is a major factor, which influences the decision to start VV-ECMO, no predictive score has shown it to be predictive of survival. Potential reasons for this include a direct effect of ECMO which reverses the adverse effects of hypoxia, bias induced by lack of information on “equally hypoxic” patients who do not receive ECMO or a type II statistical error as a result of the studies being underpowered to detect a small adverse effect from hypoxia. On the other hand, pre-ECMO direct and indirect markers of reduced compliance (e.g. high PaCO_2_, high peak inspiratory pressure, plateau pressure > 30 mmHg or pre-ECMO barotrauma evidence) were strongly associated with poor outcome in the PRESERVE, RESP and VV-ECMO mortality scores [6, 27, 56].

### Cause of respiratory failure

Aetiology is important in determining the prognosis of ECMO-treated severe ARDS. Influenza-induced ARDS was consistently associated with better outcome in the Roch et al., PRESERVE and RESP scores (70, 83 and 70% survival, respectively) [6, 27, 28]. The ECMOnet score [53], which was derived within this specific population, exhibited worse discrimination performance when it was applied in an all-cause ARDS population (area under the ROC curve of 0.60 in the external cohort validation vs 0.86 in the original cohort). With the exception of the RESP score [27], which showed that certain diagnoses such as “aspiration pneumonitis” had particularly good survival, patient numbers in most studies have been too small to detect significant relationships between other specific diagnoses and outcome.

### Prediction model limitations and performance

All scores seem to perform better compared with classical ICU severity scores [6, 27, 28, 53, 54]. However, the differences in model composition illustrate heterogeneity of the ECMO databases in terms of size, population and data collected.

Some of these scores were specifically focused on dedicated populations, which limit applicability to other ARDS diagnosis. For instance, the ECMOnet score was built on an influenza A(H_1_N_1_) ARDS cohort ventilated for less than 7 days [53], whereas Roch et al.’s score was designed for patients transferred to a referral centre for ECMO [28]. Variation in data collected in the ECMO databases influences the composition and the performance of survival prediction models, and we cannot rule out that other pre-ECMO items not collected in these databases might also impact on the prognosis. While pre-ECMO prone positioning and use of neuromuscular blockage agents are constituent parts of some scores [6, 27], other studies did not collect these variables [54–56] or found no statistical association with mortality [28]. Third, the statistical methodology used to construct the different scores is heterogeneous. All scores have used logistic regression techniques but none have employed mixed or random-effects models. Only two scores have used bootstrapping [27, 54] and only three out of seven have been validated externally [6, 27, 53] (Table 1). Fourth, because patients’ prognosis after ECMO has markedly improved over the last two decades, performance of these scores and derived predicted mortality rates might also change over time. Fifth, these models have been developed for patients already on ECMO and not validated for survival prediction in a general population of severe acute respiratory failure patients where ECMO has not (yet) been instituted. The models should therefore not be considered substitutes for clinical judgment. Lastly, caution should always be taken when using survival probabilities derived from these scoring systems to inform families and relatives on a patient’s prognosis because ECMO remains associated with devastating complications such as neurological bleeding which may occur despite favourable pre-ECMO score-based predicted survival.

## Conclusion

Although the use of ECMO for severe refractory ARDS has markedly increased since 2009, in-hospital mortality remains high (from 35 to 45%). In addition, despite major technological improvement of the devices, ECMO is still associated with numerous therapy-related complications and significant physical/psychological long-term impairment. These factors reinforce the need to perform ECMO in high volume [59] and expert referral centres with appropriate and accurate selection of patients who are mostly to obtain benefit over standard therapies. On the basis of a large development cohort, external validation and easily available web calculator (www.respscore.com), we recommend the RESP score to benchmark outcomes, to interpret variation in practice and to inform clinicians and families of likely outcomes for patients treated with ECMO for severe respiratory failure, and await with interest future prospective interventional studies to inform clinicians about when, how and on whom to perform ECMO.
